# Antimicrobial resistance and one health in the post COVID-19 era: What should health students learn?

**DOI:** 10.1186/s13756-022-01099-7

**Published:** 2022-04-11

**Authors:** Osman Kamal Osman Elmahi, Saad Uakkas, Babatunde Yusuf Olalekan, Ibrahim Abdulmumin Damilola, Oluwakorede Joshua Adedeji, Mohammad Mehedi Hasan, Ana Carla dos Santos Costa, Shoaib Ahmad, Mohammad Yasir Essar, Deborah Janine Thomson

**Affiliations:** 1 Faculty of Medicine, Ibn Sina University, Khartoum, Sudan; 2 London School of Hygiene & Tropical Medicine, Keppel Street, London, UK; 3 Faculty of Pharmaceutical Sciences, University of Ilorin, Ilorin, Nigeria; 4 Department of Biochemistry and Molecular Biology, Faculty of Life Science, Mawlana Bhashani Science and Technology University, Tangail, Bangladesh; 5 Faculty of Medicine, Federal University of Bahia, Salvador, Bahia, Brazil; 6 Department of Medicine and General Surgery, Punjab Medical College, Faisalabad, Pakistan; 7 Kabul University of Medical Sciences, Kabul, Afghanistan; 8 One Health Lessons, Arlington, VA USA

**Keywords:** Antimicrobial resistance, One Health, COVID-19, Health students, Education, Curriculum, AMR

## Abstract

Antimicrobial resistance (AMR) is a critical worldwide health issue that jeopardizes our ability to fight illnesses. However, despite being a natural phenomenon, AMR is exacerbated in the world by inappropriate administration of an antimicrobial medication such as under-use or overuse by the general population, farmers, and various health professionals. The onset of the COVID-19 pandemic has put the world in a shocking state. The pandemic exacerbated the problem of antimicrobial resistance, which was largely caused by irrational off-label use of antivirals, anthelmintics, antimalarials, and, most notably, macrolide antibiotics. As a result, monitoring the AMR progression during the pandemic has been critical. The One Health Approach is progressively becoming the most widely utilized and recommended approach in the ongoing fight against AMR. The aim of this article is to address the lack of teachings in AMR and the One Health Approach in health student training curricula, as well as to provide recommendations that can be implemented as we progress beyond the COVID-19 era.

## Introduction

The World Health Organization (WHO) considers antimicrobial resistance (AMR) to be a major global health issue that jeopardizes our ability to combat infections [1]. As of 2016, more than 700,000 deaths per annum have been attributed to the unsuccessful treatment of bacterial infections due to antimicrobial resistance [2]. It is projected that, if nothing changes in our actions, AMR could cause more than 10 million deaths per year globally by 2050 and cost the world approximately US$100 trillion in productivity loss [3]. In the United States, more than 2.8 million multidrug resistant bacterial infections occur every year, leading to over 35,000 deaths and US$20 billion in healthcare expenditures as of 2020 [3].

Although the development of AMR is a natural phenomenon, the accelerated rate in AMR can be attributed in part to inappropriate prescriptions including under-use and overuse of antibiotics by both the general public and health professionals [4, 5]. The lack of clean water and sanitation, inadequate infection prevention and control, and the inadequate use of antibiotics for animals and in agriculture only worsens the situation, by promoting the spread of resistant microbes [4]. Moreover, the transfer of antibiotic resistance genes from one resistant microorganism to another nearby naive microorganism can occur via transformation, conjugation, and transduction [6].

The COVID-19 pandemic added a burden to the existing problem of antimicrobial resistance, mainly due to the fear-induced off-label use of drugs such as antivirals, anthelmintics, antimalarials and, mainly, macrolide antibiotics such as azithromycin. In many cases of COVID-19, evidence-based medicine practices implied in selecting the most suitable pharmacotherapy in accordance with updated evidence. However, prior to the advent of antiviral drugs targeting COVID-19, selection of appropriate pharmacotherapy in developing nations was based on physicians’ judgement rather than empiric evidence demonstrated in clinical trials [7]. In the era of COVID-19, it is also even more essential to pay attention to antimicrobial resistance as issues of opportunistic infections, co-infections and multidrug resistance had been increasing in complicated COVID-19 cases [8].

According to the WHO, a holistic approach is critical to stunting antibiotic resistance. Expanding upon this, addressing and combating antimicrobial resistance requires a collaborative and multidisciplinary approach of all stakeholders, especially in a post-COVID-19 era while transferring antimicrobial resistance genes (ARGs) do not recognize “geographical, animal or human” borders [4].

In this continuing fight against antimicrobial resistance, the One Health Approach is also gradually becoming the most used and encouraged approach [4, 9]. It is of extreme importance to reduce the inappropriate utilization of antimicrobials and promote good sanitation measures, including both infection control and antibiotic stewardship practices in hospitals, pharmacies, as well as at farms; it is also of vital importance to promote other options such as available vaccines, water and sanitation hygiene, and infection prevention and control to prevent diseases before treatment is required. The One Health Approach stimulates improvement in policy [10] and regulation regarding antimicrobial use and consumption, surveillance, stewardship, infection control, sanitation, animal husbandry and alternatives to antimicrobials rather than advocating individually to combat future AMR concerns. Taking the One Health Approach, meaning a multidisciplinary team approach, could likely influence strong science policy development [10] in the realm of antimicrobial usage, monitoring, stewardship, infection control, sanitation practices in both hospital and animal hospital settings as well as an increase the chance of alternative medication development.

Therefore, reducing the over-prescription and misuse of antimicrobials requires urgent actions, and should start in the academic field, where both health professionals and health students (of all health disciplines) must be adequately trained to run antimicrobial stewardship programmes, especially using the One Health Approach. Moreover, students should be taught how to efficiently speak with policy makers as well as patients and/or their caretakers and the media to convey their knowledge in a meaningful way. This will allow them to improve future policy to combat AMR and act as health promoters to influence behavioral change within the community [10].

The present article aims to address the lack of topics about AMR and the One Health Approach in the training curriculum for health students, and to present actions that can be gradually adopted as we move into a post-COVID-19 era.

## Addressing the deficiency of antimicrobial resistance topics in health students’ education curriculum

Despite major developments and advances in antimicrobial stewardship, evident gaps of knowledge regarding antibiotic stewardship and resistance still exist, especially among health students, the future health practitioners. Despite recognizing the severity of the AMR crisis, a 2020 study has demonstrated that multiple health and professional schools have not succeeded in fully preparing students for their roles as stewards against AMR [11]. In a study conducted in China among public health undergraduates (n = 1115), there was a knowledge gap identified in relation to antimicrobial resistance [12]. In another study conducted among final year medical students (n = 357) in Europe, more than 80% of the candidates selected from the study’s medical schools had not heard about the concept of “One Health” (One Health is defined as a collaborative, and transdisciplinary approach at the local, regional, national, and global levels with the goal of achieving optimal health outcomes recognizing the interconnection between people, animals, plants, and their shared environment) and its significance in fighting AMR [5, 13]. In a 2018 study conducted among final year undergraduate paramedical students in Ethiopia (n = 323), about half of the participants have “poor knowledge” of AMR [14]. In Rwanda, it was observed in 2020 that medical students held major misconceptions regarding antimicrobial resistance despite good understanding (n = 228), with 83% being unfamiliar with the concept of antimicrobial stewardship. Furthermore, only 60% believed that antimicrobials were used excessively [15]. Among veterinary students in Nigeria who participated in a 2019 study (n = 426), an unsatisfactory level of AMR awareness was also observed with over 60% receiving knowledge scores lower than average [16].

Only one-third of final-year medical students in South Africa were confident enough to prescribe antibiotics (n = 289), by adhering to national guidelines and being familiar with antimicrobial stewardship and establishing frequent contact with infectious disease physicians. Moreover, the majority of final-year medical students (95%) appreciated that increasing the content of antimicrobial resistance in the medical curriculum is a necessity. This finding was fortified by their erroneous perception that poor hand hygiene is insignificant in contributing to antimicrobial resistance [17].

These discrepancies could be attributed to the absence of a curriculum dedicated to antimicrobial resistance; medical schools in Africa, for example, traditionally address antimicrobial resistance in multiple subjects or courses, while pharmacy degree curricula may entail a dedicated course [15]. Therefore, the majority of healthcare students have not been receiving appropriate information regarding AMR awareness. Based on the aforementioned studies, it appears that the AMR education is insufficient to prepare future healthcare professionals to combat the rising AMR crisis.

## The need for strengthening AMR training and education for health students

Since AMR is a complex global problem, there is no single way to completely combat it, so rational antimicrobial use among multiple disciplines is one of the most important strategies for preventing it [14]. The World Health Assembly adopted a global action plan on AMR in May 2015, with the first goal of the plan being to “improve awareness and understanding of antimicrobial resistance through effective communication, education and training” [9]. Achieving the main aim of ensuring the care and prevention of infectious diseases with quality-assured safe and efficient drugs would be difficult without completing this first objective and it is therefore important that healthcare students receive sufficient training in the use of antibiotics and other antimicrobials.

Antimicrobial misuse, which is one of the key factors of resistance, is greatly driven by fear- fear of being sued, fear of losing a patient, fear of the unknown. As a result, it is critical to raise awareness and attitudes among healthcare students (future healthcare professionals) about AMR's causes and control strategies, as well as related concepts. Research has suggested that providing thorough training and raising concern about AMR among healthcare students may be an efficient and motivating way to enhance potential practitioners' rational prescribing behaviors [18–21]. According to previous research and the WHO, incorporating antimicrobial stewardship into undergraduate curricula and embracing fair usage, prescribing, and dispensing is imperative for both medical and paramedical students [21–23].

## Recommendations and conclusion

Regardless of whether a health student is in veterinary medicine, allopathic medicine, paramedicine, pharmacy, or nursing school, the development and implementation of AMR and One Health topics in the university curriculum is important in equipping health professionals to fully combat AMR in their respective fields. As upcoming health practitioners, medical and allied health students and professors in universities must collaborate to develop new strategies to educate the student body. This action has shown potential to culminate in behavioral change and ensure more appropriate usage of antimicrobial drugs, especially in environments where antimicrobial administration is not adequately controlled [16].

It can also therefore be encouraged that institutions synergize to share knowledge on useful ways of training healthcare students in addition to meaningful ways to communicate AMR concerns with patients and the public. The WHO AMR Competency Framework is a suitable framework for implementation of skills-based training, knowledge sharing and developing competency as healthcare professionals in fighting the AMR crisis [24].

Also, various teaching modalities could influence the outcome of antimicrobial prescribing practices. Despite the limited impact of formal lectures and problem-based learning in teaching medical students the principles of antimicrobial stewardship, the outcome of antibiotic prescribing could be drastically improved by a re-evaluation of problem-based learning strategies, enhancing content and conduction of virtual learning platforms and development of a standardized curriculum on antimicrobial resistance [12]. Interprofessional education and team-based learning can also be a facilitator to familiarize students with the One Health concept.

Furthermore, it can also be encouraged that education and health ministries empower public health institutes and university researchers in low-income and lower middle-income countries to investigate aspects of AMR to support and fortify their National Action Plan (NAP) implementation, as they demonstrate strong potential for influencing political and professional thinking, which may culminate in continuous development of AMR modules in university curriculums [25].

Given that today's healthcare professionals will pass the metaphorical baton to tomorrow's healthcare professionals through mentorship and teaching in schools, it is critical to improve and empower healthcare students by providing rigorous AMR education and training today. This will aid in the development of today’s young health professionals as well as future professionals with the necessary skills to prevent and tackle the spread of AMR with appreciation and knowledge of the One Health Approach.

## Data Availability

Not applicable.

## Abbreviations

WHO: World Health Organization
AMR: Antimicrobial resistance
COVID-19: Coronavirus disease 2019
